# Chloride intracellular channel (CLIC) protein function in S1P-induced Rac1 activation requires membrane localization of the C-terminus, but not thiol-transferase nor ion channel activities

**DOI:** 10.3389/fcell.2025.1565262

**Published:** 2025-04-01

**Authors:** De Yu Mao, Jordan J. Jesse, Daniel D. Shaye, Jan Kitajewski

**Affiliations:** ^1^ Department of Physiology and Biophysics, University of Illinois at Chicago, Chicago, IL, United States; ^2^ Graduate Education in Biomedical Sciences program, University of Illinois at Chicago, Chicago, IL, United States; ^3^ Center for Cardiovascular Research, University of Illinois at Chicago, Chicago, IL, United States; ^4^ University of Illinois Cancer Center, University of Illinois at Chicago, Chicago, IL, United States

**Keywords:** CLIC1, CLIC4, RhoA, Rac1, sphingosine-1-phosphate, S1P receptor, endothelium

## Abstract

We have established a novel and evolutionarily-conserved function for chloride intracellular channel proteins (CLICs) in regulating Rho/Rac GTPases downstream of G protein-coupled receptors (GPCRs). Endothelial CLIC1 and CLIC4 are rapidly and transiently re-localized from the cytoplasm to the plasma membrane in response to the GPCR ligand sphingosine-1-phosphate (S1P), and both CLICs are required to activate Rac1 in response to S1P, but how they perform this function remains unknown. Biochemical studies suggest that CLICs act as non-specific ion channels and/or as glutathione-S-transferases, dependent on N-terminal features, *in vitro.* Here we investigate CLIC functional domains and membrane localization requirements for their function in S1P-mediated Rac1 signaling. Structure-function analyses of CLIC function in endothelial cells demonstrate that CLIC1 and CLIC4-specific functions reside at their C-termini, and that the CLIC4 N-terminus encodes determinants required for S1P-induced re-localization to the plasma membrane but is dispensable for S1P-induced Rac1 activation when the C-terminus is localized to the plasma membrane via a heterologous signal. Our results demonstrate that the postulated ion channel and thiol-transferase activities of CLICs are not required for Rac1 activation and suggests that sequences in the CLIC C-termini are critical for this function. Given the importance of S1P signaling in vascular biology and disease, our work establishes a platform to further our understanding of the membrane-localized proteins required to link GPCR activity to Rho/Rac regulation.

## Introduction

Chloride intracellular channels (CLIC) are a family of evolutionarily conserved proteins, with vertebrate genomes encoding six paralogs, that play important roles in development and disease. Mouse *Clic5a* has been implicated in kidney development and function (Wegner et al., 2010; Tavasoli et al., 2016a; Tavasoli et al., 2016b), while global *Clic4* knockout mice have vascular defects (Chalothorn et al., 2009; Ulmasov et al., 2009), predisposition to pulmonary hypertension (Wojciak-Stothard et al., 2014), predisposition to kidney injury (Edwards et al., 2014), and skin and corneal wound healing defects (Padmakumar et al., 2012), and we recently used a conditional allele to show that endothelial *Clic4* regulates lung vascular permeability (Kleinjan et al., 2023). In addition, several studies have implicated human *CLIC1* in Alzheimer’s disease (Novarino et al., 2004; Carlini et al., 2020) and CLICs in cancer (Suh et al., 2007; Macpherson et al., 2014; Wang et al., 2014; Flores-Tellez et al., 2015; Peretti et al., 2015; Setti et al., 2015; Hernandez-Fernaud et al., 2017; Sanchez et al., 2022).

Despite their clear role in development and disease, the physiologically-relevant molecular functions of CLICs have remained elusive. Vertebrate CLICs can exist as nuclear, cytoplasmic, and/or membrane-associated forms, and different extracellular signals or physiological conditions can promote re-localization between these different cellular compartments (Novarino et al., 2004; Ponsioen et al., 2009; Malik et al., 2010; Gurski et al., 2015; Lecat et al., 2015; Carlini et al., 2020; Mao et al., 2021; Kleinjan et al., 2023). Although some CLICs transiently, or constitutively, reside at membranes, chloride channel activity under physiological conditions has been challenging to establish (reviewed in Argenzio and Moolenaar, 2016), raising the question as to whether their postulated ion channel activity is required for their physiological functions. In addition, the crystal structure of invertebrate and human CLICs (Littler et al., 2004; Littler et al., 2005; Cromer et al., 2007; Littler et al., 2008) revealed that these proteins resemble the omega family of glutathione-S-transferases (Ω-GSTs), and some purified CLICs exhibit GST activity *in vitro* (Al Khamici et al., 2015; Alghalayini et al., 2023), but whether this GST-like activity is required for physiological CLIC function is also an open question. Recent work from us, and others, identified a role for CLICs in G protein-coupled receptor (GPCR) and heterotrimeric G-protein-regulated Rho and Rac signaling that is conserved from *C. elegans* to humans (Tavasoli et al., 2016b; Mao et al., 2021; Arena et al., 2022; Kleinjan et al., 2023). However, whether the GST and/or ion channel activities are relevant to CLIC function in GPCR and Rho-family GTPase signaling remains unknown, and is a question we address here.

**FIGURE 1:**
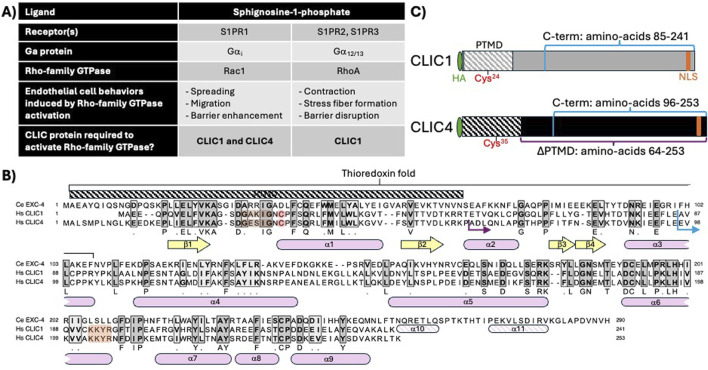
CLIC1 and CLIC4 in endothelial S1P signaling, and CLIC sequence and structural features relevant to this study. **(A)** description of shared and unique functions for CLIC1 and CLIC4 in endothelial S1P signaling, as defined in Mao et al. (2021). **(B)** Protein sequence alignment of *C. elegans* EXC-4, and human CLIC1 and CLIC4 indicating regions of amino-acid identity (dark grey shading) and similarity (light grey shading). Conserved beta sheets (yellow arrows) and alpha helices (pink rounded boxes). The N-terminal putative transmembrane domain (PTMD) and conserved thioredoxin fold are highlighted. Note that the N-terminal cysteine required for thiol-reductases activity (red), the cholesterol-binding GxxxG motif (brown), and C-terminal nuclear localization signal (orange) found in human CLIC1 and four are not present in *C. elegans* EXC-4, while the *C. elegans* protein has a C-terminal extension with additional alpha helices (α10, α11). **(C)** cartoon representation of HA-tagged full-length CLIC1 and CLIC4 constructs. The C-terminal fragments swapped in chimeric constructs (Figure 2) are denoted in blue, and the truncated (∆PTMD)CLIC4 construct (Figures 3, 4) is denoted in purple [the starting protein sequence for chimeric and truncated constructs are shown as blue and purple arrows, respectively, in panel **(B)**].

CLIC1 and CLIC4 are expressed in the endothelium (Tung et al., 2009; Tung and Kitajewski, 2010; Mao et al., 2021), and we previously used human umbilical vein endothelial cells (HUVEC) to establish that they are required for cell viability, migration, and angiogenic behaviors (Tung et al., 2009; Tung and Kitajewski, 2010). We also found that activation of the sphingosine-1-phosphate (S1P) and thrombin GPCR pathways induces transient re-localization of CLIC1 and/or CLIC4 to the plasma membrane (hereafter PM), and that CLIC1 and CLIC4 act in distinct GPCR cascades to regulate RhoA and/or Rac1 activation. Specifically CLIC1 and CLIC4 function in sphingosine-1-phosphate receptor (S1PR) signaling in HUVEC (Mao et al., 2021), while CLIC4 functions in the thrombin/protease activated receptor (PAR) pathway in HUVEC and *in vivo* (Kleinjan et al., 2023). Here we evaluate CLIC function in S1P signaling (summarized in Figure 1A). S1P binding to S1PR1 activates its coupled Gα subunit, Gα_i_, which in turn activates Rac1, promoting cell spreading, migration, and endothelial barrier enhancement (Sugimoto et al., 2003; Kono et al., 2004; Reinhard et al., 2017), while S1P binding to S1PR2 and S1PR3 activates RhoA, via Gα_12/13_, leading to stress-fiber formation, cell contraction and endothelial barrier disruption (Lee et al., 1999; Miura et al., 2000; Blaho and Hla, 2011; Reinhard et al., 2017). We found that S1P induces a rapid and transient re-localization of CLIC1 and CLIC4 to the PM, and that both of these CLICs are required for S1P-induced Rac1 activation, while only CLIC1 is required for S1P-induced RhoA activation (Mao et al., 2021). Notably, CLIC1 and CLIC4 have non-overlapping functions, as CLIC1 overexpression did not rescue the Rac1 activation defect caused by *CLIC4* knockdown, nor did CLIC4 overexpression rescue the Rac1 or RhoA activation defects caused by *CLIC1* knockdown (Mao et al., 2021).

The goals of this study are to determine which domain(s) mediate CLIC function in S1P-induced Rac1 activation. We first show that the CLIC1 and CLIC4 C-termini define their specific functions in Rac1 activation. As *Clic4* functions *in vivo* have been well-established, we carried out further structure-function studies of CLIC4 and found that the N-terminus is required for S1P-induced re-localization to the PM, consistent with the previously-described function for an N-terminal putative transmembrane domain (PTMD) in EXC-4 localization in *C. elegans* (Berry et al., 2003; Berry and Hobert, 2006), and with electrophysiology and cell culture results showing that the CLIC4 PTMD promotes membrane localization (Singh and Ashley, 2007; Ponnalagu et al., 2022). Finally, we show that replacing the CLIC4 PTMD with a heterologous PM-targeting motif (the myristylation (myr) signal of the kinase Lck. Bijlmakers et al., 1997; Zlatkine et al., 1997) induces constitutive PM localization and restores the ability of N-terminally-truncated CLIC4 to promote S1P-induced Rac1 activation. Given that the myr signal does not possess GST activity nor does it form ion channels, our results demonstrate that these CLIC activities are dispensable for CLIC4 function in S1P-induced Rac1 activation, and support a model where CLIC C-termini function at the membrane to facilitate activation of Rho-family GTPases.

## Results

### CLIC1 and CLIC4 functions in S1P-induced Rac1 activation are specified by their C-termini

Structure-function studies in *C. elegans* showed that the first 66 N-terminal amino-acids of EXC-4 are necessary and sufficient for PM localization (Berry et al., 2003; Berry and Hobert, 2006), defining a putative transmembrane domain (PTMD), which encompasses two ß-sheets flanking an α-helix, structural elements that are conserved in CLIC1 and CLIC4 (Figure 1B. Harrop et al., 2001; Littler et al., 2005; Littler et al., 2008), and a cholesterol-binding motif that facilitates CLIC interaction with membranes (Figure 1B. Valenzuela et al., 2013; Al Khamici et al., 2016; Hossain et al., 2016; Hossain et al., 2019). The PTMD resides within the “thioredoxin fold”, which encodes the thiol-transferase activity of Ω-GSTs that is dependent on a catalytic cysteine found at the start of the first alpha helix (α1, red in Figure 1B, C). The overlap between the PTMD and the thioredoxin fold, combined with biochemical and biophysical studies (Littler et al., 2004; Singh and Ashley, 2006; Goodchild et al., 2009; Goodchild et al., 2010; Al Khamici et al., 2015; Al Khamici et al., 2016; Hare et al., 2016; Hossain et al., 2019; Alghalayini et al., 2023), has led to a model where redox conditions and GST-like activity of CLICs induces a conformational change that exposes the PTMD, resulting in PM insertion of the N-terminus of CLIC proteins. However, the cysteine required for GST activity is absent from *C. elegans* EXC-4 (Figure 1B), which constitutively localizes to the PM (Berry et al., 2003) and which we showed also regulates Rho/Rac signaling in *C. elegans* (Arena et al., 2022). Therefore, questions remain as to whether the postulated GST or channel activities are indeed required for EXC-4/CLIC function in Rho-family signaling,

We previously established functional assays for CLIC1 and CLIC4 by measuring both S1P-induced activated Rac1 using the G-LISA assay (see Methods), and S1P-induced, and Rac1-dependent, strengthening of the endothelial barrier formed by HUVEC monolayers (Garcia et al., 2001; Reinhard et al., 2017), as assessed via a trans-endothelial electrical resistance (TEER) assay (see Methods). HUVEC infected with lentivirus expressing short-hairpin RNAs (see Methods) targeting *CLIC1* or *CLIC4* (hereafter *CLIC1* ^*KD*^ or *CLIC4* ^*KD*^) are deficient in S1P-induced Rac1 activation and barrier enhancement when compared to controls (Mao et al., 2021), while co-infection with lentivirus expressing HA-tagged full-length CLIC1 or CLIC4 led to selective rescue of these phenotypes: HA-CLIC1 rescued phenotypes caused by *CLIC1* ^*KD*^ but not *CLIC4* ^*KD*^, while HA-CLIC4 rescued *CLIC4* ^*KD*^ but not *CLIC1* ^*KD*^ (Mao et al., 2021). These results showed that there is specificity to CLIC function in S1P-induced Rac1 activation, and that both CLICs are required, non-redundantly, to facilitate the S1PR1-Gαi branch of the pathway to activate Rac1.

**FIGURE 2:**
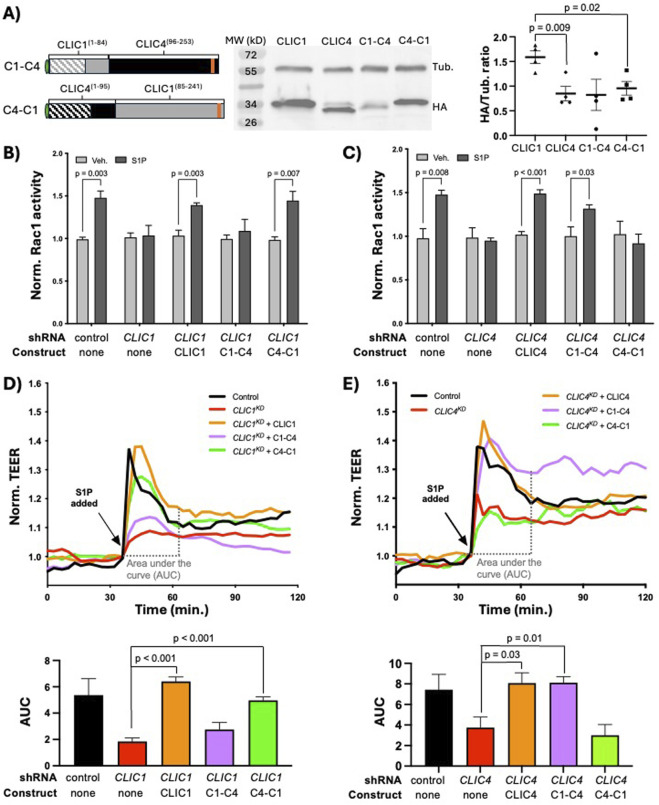
CLIC1 and CLIC4-specific functions in S1P signaling are encoded by their C-termini. **(A)** cartoon representation of the C1-C4 and C4-C1 chimeras, and western blot analysis and quantification (n = 4) of their expression in HUVEC (see Methods). In panels **(B)** and **(C)** active Rac1 measured by G-LISA (see Methods). Here, and in subsequent figures showing G-LISA results, each bar represents the mean of n ≥ 3 replicates, and error bars represent standard deviation (SD). Significance between vehicle control (Veh.) and S1P-treated cells was calculated via two-tailed unpaired t-tests. **(B)** full-length CLIC1 and the C4-C1 chimera rescue the Rac1 activation defect caused by *CLIC1* ^*KD*^, while **(C)** full-length CLIC4 and the C1-C4 chimera rescue the Rac1 activation defect caused by *CLIC4* ^*KD*^. In panels **(D, E)**, and in subsequent figures showing TEER results, each trace represents the mean trans-endothelial electrical resistance (TEER) measurement of n ≥ 3 samples, normalized to the TEER value at time of S1P addition (see Methods). To quantify TEER results, the area under the curve (AUC) ± standard error of the mean (SEM) from the time of S1P addition (∼30min after beginning of resistance measurements) until time of maximal recovery to baseline (∼30 min after S1P addition in controls) was calculated for each condition (see Methods), and results are shown in the accompanying bar graphs. Significance was calculated using unpaired two-tailed t tests. **(D)** full-length CLIC1 and the C4-C1 chimera rescue the barrier defect caused by *CLIC1* ^*KD*^, while **(E)** full-length CLIC4 and the C1-C4 chimera rescue the TEER defect caused by *CLIC4* ^*KD*^.

Because the EXC-4 N-terminus promotes membrane accumulation but does not by itself rescue *C. elegans exc-4* null mutants (Berry et al., 2003; Berry and Hobert, 2006), we hypothesized that the CLIC1 and CLIC4 N-termini primarily mediate localization, while CLIC-specific signaling functions are encoded by their C-termini. To test this hypothesis, we created HA-tagged chimeras (see Methods, and Figures 1C, 2A), where the N-terminus of CLIC1 was fused to the C-terminus of CLIC4 (C1-C4), and vice-versa (C4-C1), and assessed their expression in HUVEC via western blotting. Notably, we found that CLIC1 was consistently expressed at significantly higher levels than CLIC4 (Figure 2A), and because both CLICs are expressed from the same lentiviral vector (see Methods), this difference likely reflects differential post-transcriptional regulation between CLIC1 and CLIC4. Western analysis of chimeric proteins (Figure 2A) showed bands of the expected sizes with variable levels of expression. We next tested the ability of these chimeras to rescue *CLIC1* ^*KD*^ or *CLIC4* ^*KD*^ phenotypes in HUVEC, and found that full-length CLIC1 and C4-C1, but not the C1-C4 chimera, rescued *CLIC1* ^*KD*^ defects (Figure 2B, D). Conversely, *CLIC4* ^*KD*^ defects were significantly rescued by full-length CLIC4 and by C1-C4, but not by the C4-C1 chimera (Figure 2C, E). These results demonstrate that the CLIC1 and CLIC4 C-termini provide specificity to CLIC function in endothelial S1P-induced Rac1 activation.

### The PTMD is necessary for S1P-induced CLIC4 membrane re-localization and Rac1 activation.

**FIGURE 3:**
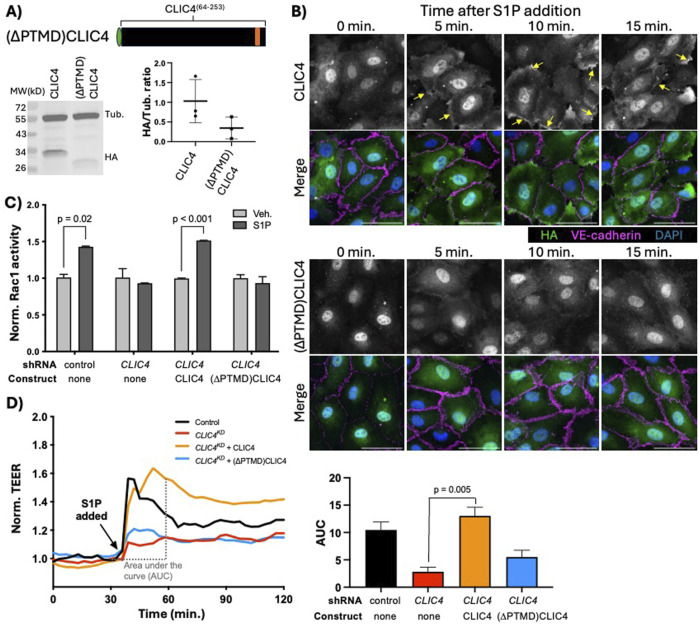
The N-terminal putative transmembrane domain (PTMD) is required for CLIC4 re-localization and Rac1 activation in response to S1P. **(A)** cartoon representation of the HA-tagged (∆PTMD)CLIC4 construct, and western blot analysis and quantification of its expression in HUVEC (see Methods), as compared to full-length HA-CLIC4. **(B)** Immunofluorescence of full-length HA-tagged CLIC4 (top) and (∆PTMD)CLIC4, and their localization after treatment with S1P. Yellow arrows denote PM localization. Scale bar represents 50 µm in all panels. **(C)** the (∆PTMD)CLIC4 construct does not rescues the Rac1 activation defect caused by *CLIC4* ^*KD*^, as assessed by G-LISA. **(D)** the (∆PTMD)CLIC4 construct does not rescue the TEER defect caused by *CLIC4* ^*KD*^.

To further probe the function of the CLIC N- and C-termini, we generated a truncated CLIC4 lacking the PTMD (hereafter (∆PTMD)CLIC4. Figures 1B, 3A) and expressed this truncated protein in HUVEC. We assessed expression via western blotting and detected a band of the expected size (Figure 3A). Expression of (∆PTMD)CLIC4 was typically lower than that of full-length protein, but this difference did not achieve statistical significance, possibly due to expression variability between experiments (Figure 3A). Both constructs showed nuclear and cytoplasmic accumulation prior to S1P treatment (Figure 3B), and noticeable accumulation of full-length CLIC4 was observed near the cell cortex (outlined by VE-cadherin) within 5 min, and remained at 10 and 15 min, after S1P treatment (yellow arrows in top rows of Figure 3B). In contrast we did not observe similar re-localization of the (∆PTMD)CLIC4 construct (bottom rows of Figure 3B). Thus, the CLIC4 PTMD is necessary for re-localization to the PM in response to S1P. We next assessed (∆PTMD)CLIC4 function and found that this protein did not rescue the Rac1 activation (Figure 3C) or TEER defects (Figure 3D) caused by *CLIC4* ^*KD*^. This lack of function suggests that either the lower levels of (∆PTMD)CLIC4 expression precludes efficient rescue, that the PTMD encodes a function required for Rac1 activation, or that PTMD-mediated re-localization of CLIC4 to the PM is necessary for its function in S1P-induced Rac1 activation.

### A membrane-localized form of the CLIC4 C-terminus functions in S1P-induced Rac1 activation

**FIGURE 4:**
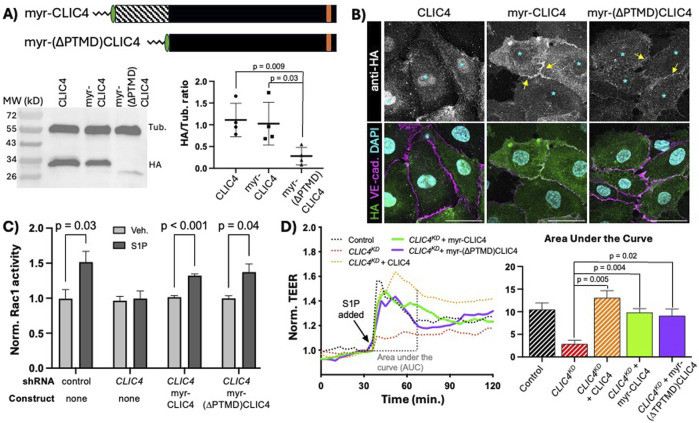
Membrane localized CLIC4 C-terminus is sufficient to promote Rac1 activation in response to S1P. **(A)** cartoon representation of HA-tagged full-length myristylated (myr) CLIC4 and myr-(∆PTMD)CLIC4, lacking the N-terminal putative transmembrane domain. Western blot analysis and quantification of expression in HUVEC (see Methods), shows lower expression of myr-(∆PTMD)CLIC4 as compared to full-length myr-CLIC4. **(B)** Immunofluorescence showing accumulation of HA-tagged full-length CLIC4 (first column), myr-CLIC4 (second column) and myr-(∆PTMD)CLIC4 (third column) at steady state (no S1P). Note that both myristylated constructs display accumulation at the membrane (yellow arrows), and decreased nuclear accumulation (cyan asterisks) when compared to wildtype full-length CLIC4. Scale bar represents 50 µm in all panels. **(C)** both the full-length myr-CLIC4 and the myr-(∆PTMD)CLIC4 constructs rescue the Rac1 activation defect caused by *CLIC4* ^*KD*^, as assessed by G-LISA. **(D)** both the full-length myr-CLIC4 and the myr-(∆PTMD)CLIC4 constructs rescue the TEER defect caused by *CLIC4* ^*KD*^. Dashed lines and bars (Control, *CLIC4* ^*KD*^, and *CLIC4* ^*KD*^ + CLIC4) are the same data shown in Figure 3D.

We sought to determine if the prime function of the CLIC4 N-terminus is to facilitate PM localization, and, based upon our finding that the C-terminus of CLICs confer signaling specificity (Figure 2), we also posited that the C-terminus may be the domain responsible for CLIC4-mediated Rac1 activation. To address these questions, we used a heterologous membrane-localization signal, the myristylation (myr) signal of the kinase Lck (Bijlmakers et al., 1997; Zlatkine et al., 1997), to induce constitutive membrane localization of ectopically expressed CLIC4. We generated two new constructs with myr signal appended to the N-terminal HA-tag: a full length myr-CLIC4, to assess effects of the myr signal on full-length CLIC4 expression and function, and a truncated myr-(∆PTMD)CLIC4. We assessed expression of these constructs via western blotting, finding single bands of the expected size, noting that there was no significant difference in expression levels between full-length CLIC4 and myr-CLIC4, while the the truncated form consistently and significantly dysplaed lower expression than the full-length proteins (Figure 4A). Notably, both myr-CLIC4 and myr-(∆PTMD)CLIC4 were constitutively localized to the PM in the absence of S1P (Figure 4B), indicating that, as expected, the myr signal confers constitutive PM accumulation. Importantly, myr-CLIC4 the Rac1 activation (Figure 4C) and TEER defects (Figure 4D) caused by *CLIC4* ^*KD*^. Thus, we conclude that the myr signal does not interfere with CLIC4 function. We also found that myr-(∆PTMD)CLIC4 rescues *CLIC4* ^*KD*^, leading us to conclude that the C-terminal domain encodes the function(s) of CLIC4 required for Rac1 activation in response to S1P. Moreover, because the PTMD encodes sequences necessary for GST-like and ion channel activities, rescue by myr-(∆PTMD)CLIC4 establishes that GST and ion channel functions are not necessary for CLIC4 function in S1P-induced Rac1 activation.

## Discussion

CLICs have emerged as conserved players in Rho-family GTPase signaling in contexts as diverse as the *C. elegans* excretory canal (Arena et al., 2022), mouse kidney podocytes (Tavasoli et al., 2016b), mouse vasculature (Ulmasov et al., 2009; Kleinjan et al., 2023), and human endothelial cells (Mao et al., 2021; Kleinjan et al., 2023). The molecular function of CLICs has long-remained mysterious, with structural and biochemical studies suggesting they function as channel and/or thiol-transferases [reviewed in Argenzio and Moolenaar (2016)]. However, questions remained as to the relevance of these CLIC activities for their role in GPCR and Rho-family GTPase signaling. We addressed this issue by undertaking structure-function studies and our results demonstrate that the signaling functions of CLIC1 and CLIC4 are encoded by their C-termini, while the N-terminus is not required for signaling and mainly plays a role in promoting PM accumulation upon GPCR activation. Our results are consistent with findings in *C. elegans* showing that the N-terminal PTMD is necessary and sufficient for PM accumulation, and also required for EXC-4 function (Berry et al., 2003; Berry and Hobert, 2006), and with electrophysiology and cell culture results showing that the CLIC4 PTMD spans across lipid bilayers and mediates membrane localization (Singh and Ashley, 2007; Ponnalagu et al., 2022). An open question is whether the CLIC4 PTMD inserts into, and spans, the PM in response to S1P to facilitate Rac1 signaling, and whether membrane insertion is required for this function. Our results showing that the CLIC4 PTMD can be replaced by a myr signal (which does not span the membrane) suggests that PM insertion is not necessary for CLIC4 function in Rac1 activation, but does not rule out the possibility that full insertion might potentiate or increase signaling capacity, or that membrane insertion of CLIC4, or CLIC1, may be required in other signaling contexts. Our results also suggest differential post-transcriptional regulation of CLIC1 and CLIC4 protein levels, and we found that CLIC4 constructs lacking the PTMD appear to express at lower levels than full-length protein, even when tethered to the PM, suggesting that the CLIC4 PTMD may encode determinants that promote stability and/or counteract degradation signals located at the C-terminus.

Rho-family GTPase activity is precisely controlled by regulating their PM localization, and the PM localization of activators (guanine-exchange factors, or RhoGEFs) and inhibitors (GTPase-activating proteins, or RhoGAPs) (reviewed in Mosaddeghzadeh and Ahmadian, 2021). Our finding that PM accumulation of the CLIC4 C-terminus is required for Rac1 activation in response to S1P suggests a model where CLIC C-termini activate Rho-family GTPases either directly, or as part of a complex with RhoGEFs and/or RhoGAPs. If CLICs directly activate Rho-family GTPases, we might expect that constitutive recruitment of full-length, or just the C-terminus of, CLIC4 to the PM would be sufficient to activate Rac1. However, we did not observe Rac1 activity over basal levels in cells expressing either myr-CLIC4 or myr-(∆PTMD)CLIC4 in the absence of S1P (Supplementary Figure S1), indicating that PM-localized CLIC4 is necessary, but not sufficient, to activate Rac1.

CLICs have been implicated in many cell processes, including vesicle trafficking, secretion, cell polarity, apoptosis, proliferation, cytokinesis, and motility (Tonini et al., 2000; Berry et al., 2003; Ulmasov et al., 2009; Salles et al., 2014; Wei et al., 2015; Zhao et al., 2015; Chou et al., 2016), and most, if not all, of these processes are regulated by Rho-family GTPases (Jaffe and Hall, 2005). Therefore, defining the molecular mechanisms by which CLICs regulate Rho/Rac activity has the potential to define novel means by which to modulate GPCR-Rho/Rac signaling. We expect that continued structure-function studies to elucidate C-terminal sequences required for CLIC function, combined with discovery of CLIC-interacting proteins required for function, will reveal the novel and conserved molecular mechanisms by which CLICs regulate Rho-family GTPase activity.

## Materials and methods

### Primary cells and cell culture

Pooled HUVECs from different donors were either directly isolated from human umbilical cords following established protocols (Jaffe et al., 1973) or purchased (Lonza, Cat# C2519A). HUVEC were grown in EGM-2 Endothelial Cell Growth Media (Lonza), including all supplements provided, on culture dishes coated with rat tail type I collagen (Corning). 293T cells were acquired from the American Type Culture Collection and maintained in high-glucose Dulbecco’s modified Eagle’s medium (Gibco) with 10% heat-inactivated fetal bovine serum (HI-FBS) and 0.01% penicillin-streptomycin. Unless otherwise noted, cells were cultured under standard conditions in a humidified incubator at 37°C, 5% CO_2_.

### RNAi-mediated CLIC1 and CLIC4 knockdown constructs

Validated control (scrambled) shRNA, and human CLIC1 and CLIC4 shRNA-expressing lentiviruses, based on the pLKO.1 vector backbone (Moffat et al., 2006), from Sigma-Millipore were as previously-described (Tung et al., 2009; Tung and Kitajewski, 2010; Mao et al., 2021; Kleinjan et al., 2023).

### Tagged CLIC1 and CLIC4 constructs

Full-length HA-tagged CLIC1 and CLIC4 plasmids were previously described (Mao et al., 2021), and the derived chimeric and truncated variants used here were made using standard molecular cloning techniques, and DNA sequencing was performed to verify the correct sequence before experiments (details available upon request). All HA-tagged constructs were cloned into the pCCL lentivirus vector (Dull et al., 1998) for delivery and expression in HUVEC.

### Lentivirus-mediated stable overexpression and/or knockdown in HUVEC

To perform knockdown and/or overexpression in HUVEC, a lentiviral infection system was utilized. For lentiviral gene transfer, 293T cells were transfected using the calcium phosphate approach with the following combination of plasmids: 3 μg of pVSVG, 5 μg of pMDLg/pRRE, 2.5 μg of pRSV-Rev, and 10 μg of the overexpression (pCCL-based) and/or knockdown (pLKO.1-based) vectors of interest. Transfected 293T cells were allowed to produce lentivirus and the supernatant was collected 48 h post transfection. The supernatant collected was passed through a 0.45 μm filter and then added onto HUVEC. A single round of infection was performed for 24 h s. The primary cells were allowed to express shRNA or overexpression constructs for at least 48 h before experiments. A red-flourescent construct (pCCL-RFP) was included in all experiments to assess infection efficiency.

### Reagents and antibodies

Sphignosine-1-phosphate (S1P) was obtained from Enzo Life Sciences (BML-SL140). Antibodies to human CLIC1 (Abcam ab28722, Rabbit, 1:250), human CLIC4 (Novus Biologicals NBP1-85574, Rabbit, 1:250), HA (GenScript A01244, Mouse, 1:500), tubulin (Sigma-Aldrich T6074, 1:5000), and VE-cadherin (Abcam ab33168, Rabbit, 1:250) were used for immunoblotting and immunofluorescence as described below.

### Immunoblotting

HUVECs were washed with cold PBS, and lysates were collected with TENT lysis buffer (50 mM Tris pH 8.0, 2 mM EDTA, 150 mM NaCl, and 1% Triton X-100) containing a protease inhibitor cocktail (EMD Chemicals Inc.). Protein lysates were collected by centrifugation at 14000rpm for 10 min. Lysates were boiled at 95°C for 5 min with addition of sample buffer containing SDS and β-mercaptoethanol. Protein concentrations were measured using the Bradford protein assay (Bio-Rad Laboratories). Volumes were adjusted to ensure equal amounts of protein loading. SDS-PAGE was performed for 1 h at 150V, followed by wet transfer of proteins onto a nitrocellulose membrane for 1 h at 100 V. Blocking of the nitrocellulose membrane was with 5% BSA in TBS-Tween solution for 1 h. Primary antibody incubation was done in 2.5% BSA overnight at 4°C, and secondary antibody incubation was done the next day for 2 h at room temperature. The membrane was developed using Clarity™ Western ECL Substrate (Bio-Rad) and protein bands were observed. Densitometry analysis was performed with GelAnalyzer 19.1 by Istvan Lazar Jr., PhD and Istvan Lazar Sr., PhD, CSc (available at www.gelanalyzer.com).

### Immunofluorescence

Cells were plated on 8-well collagen-coated chamber slides (Ibidi). 50,000 cells were seeded onto each well overnight. The next day cells were serum starved for 3 h prior to S1P treatment. 1μM S1P for various times points was used to treat HUVECs, followed by fixation with 4% PFA for 10 min. Cells were then washed 3 times with PBS and blocked with 3% BSA and 0.1% TritonX-100. Primary antibodies were used at the listed dilutions in blocking solution and incubated overnight at 4°C. Cells were then washed with 1XPBS, 3 times for 5 min each and incubated with secondary antibody Alexa Fluor 488 (green) or Alexa Fluor 647 (far-red) at 1:1000 in blocking solution for 2 h. After washing cells 3 times after secondary antibody incubation, cells were mounted using VectaShield with DAPI (Vector Labs). Slides were imaged using Airyscan confocal microscopy with a Zeiss laser scanning microscope (LSM800). Images analysis was done with ZEN software under the same acquisition setting for among all cell lines in each experiment.

### Rac1 G-LISA activation assay

Assays were performed using a Rac1 (Cytoskeleton, BK128) G-LISA Activation Assay Kits. HUVECs were serum-starved in EGM-2 with 1% serum overnight and with serum-free EBM-2 for an additional 3 h the following day. The cells were then stimulated with 1 μM S1P for Rac1 activation. Cell lysates were harvested and snap-frozen in liquid nitrogen. The assay was then performed based on the manufacturer’s protocol.

### Trans-endothelial electrical resistance (TEER) assay

An ECIS array plate (Applied Biophysics) containing circular 250 μm diameter active electrodes connected in parallel on a common gold pad was coated with rat tail type I collagen (Corning). HUVEC cells were seeded at 50,000 cells per well and allowed to grow overnight. Cells were serum starved (EBM-2, Lonza) for 2 h, followed by a 30-min baseline resistance stabilization with the Electrical Cell-Substrate Impedance Sensing (ECIS) system (Applied Biophysics, model 1600R). 1μM S1P or BSA vehicle control were administered, and trans-endothelial resistance was monitored at a frequency of 4000 Hz with measurements taken at 3-min intervals for 24 h. Quantifications were performed by area under the curve analysis using GraphPad Prism software.

### Statistics

All experiments were repeated at least three times, and statistical tests were performed using GraphPad Prism software. Area under the curve averages and standard error of the mean (SEM) were computed using the Gagnon method, which produces a single value, so individual AUC measurements for each replicate are not determined. Unless otherwise noted, unpaired two-tailed t tests were used, and p < 0.05 were considered significant.

## Data Availability

The datasets and reagents generated for this study will be made available by the authors upon request, without undue reservation.
