# Supplementary figures and images for: Chloride intracellular channel (CLIC) protein function in S1P-induced Rac1 activation requires membrane localization of the C-terminus, but not thiol-transferase nor ion channel activities
Source: Front Cell Dev Biol. 2025 Apr 1;13:1565262. doi: 10.3389/fcell.2025.1565262 (PMC11996907; doi:10.3389/fcell.2025.1565262)

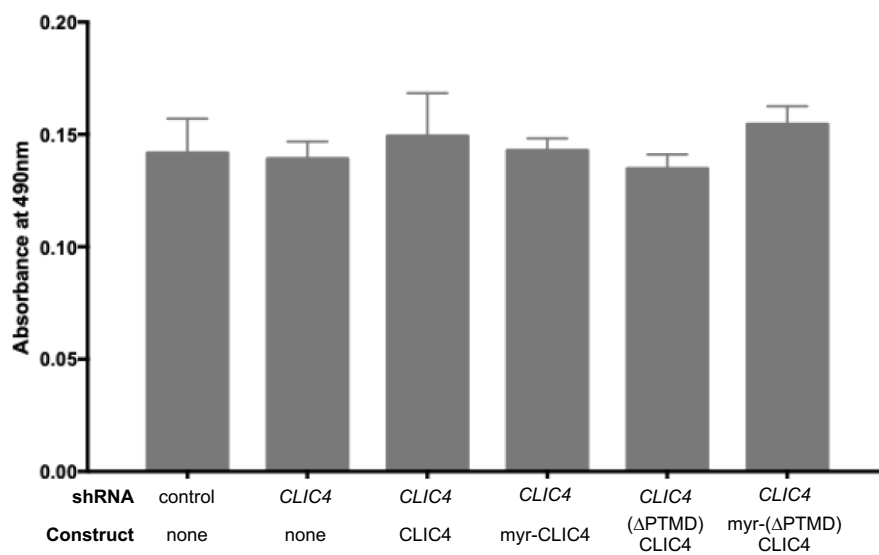

**Supplemental Figure S1:** Basal Rac1 activity is not increased by membrane tethered CLIC4

Supplement: Supplementary file 1 [file DataSheet1.pdf]
